# Threshold value of anti-Mullerian hormone for the diagnosis of polycystic ovary syndrome in Chinese women

**DOI:** 10.1371/journal.pone.0203129

**Published:** 2018-08-28

**Authors:** Chao-Yan Yue, Lou-kai-yi Lu, Meng Li, Qian-Lan Zhang, Chun-Mei Ying

**Affiliations:** 1 Department of Laboratory Medicine, Obstetrics and Gynecology Hospital of Fudan University, Shanghai, China; 2 Department of Ultrasound, Obstetrics and Gynecology Hospital of Fudan University, Shanghai, China; John Hopkins University School of Medicine, UNITED STATES

## Abstract

**Objective:**

We intended to establish the threshold for anti-Mullerian hormone (AMH) in the diagnosis of polycystic ovary syndrome (PCOS) in China.

**Methods:**

A total of 771 women (653 with PCOS and 118 healthy controls) were enrolled. The serum AMH, follicle-stimulating hormone (FSH), luteinizing hormone (LH), FSH/LH, prolactin, estradiol, testosterone (T), dehydroepiandrosterone sulfate (DHEA-S), sex hormone-binding globulin (SHBG), 17α-OH progesterone (17α-OHP), fasting insulin (INS), fasting glucose, free androgen index (FAI%) and homeostasis model assessment for insulin resistance (HOMA-IR) index were analyzed, and the diagnostic utility of AMH, LH/FSH, T and INS was established using receiver operator characteristic (ROC) curves. With AMH, LH/FSH, T and INS as independent variables, a logistic regression model was established, and the ROC curve for combined detection was fitted with the probability value of the model.

**Results:**

The serum level of FSH, LH, LH/FSH, AMH, FAI%, 17α-OHP, fasting INS, T, SHBG, DHEA-S and HOMA-IR were altered in the PCOS patients. The best compromise between sensitivity and specificity was found at an AMH cut-off level of 8.16 ng/ml and 5.89 ng/ml for the age groups 20–29 and 30–39 years, with the corresponding area under the curve being 0.846 and 0.865 respectively. The area under the ROC curve for combined detection was 0.951, which was significantly greater than that of each index. Finally, the concentration of AMH was associated with FSH, LH, LH/FSH, T, and ovarian volume in PCOS patients.

**Conclusion:**

The optimal AMH diagnostic threshold for PCOS was 8.16 ng/ml (20–29 years) and 5.89 ng/ml (30–39 years) in the Chinese population of this study. Moreover, serum AMH, LH/FSH, T and INS could be used in combination to improve the diagnostic specificity and sensitivity for the detection of PCOS.

## 1. Introduction

Polycystic ovary syndrome (PCOS) is a common reproductive endocrine disease that is prevalent in 6% of women of childbearing age [1]. Polycystic ovary syndrome (PCOS) is a common endocrine disease that is clinically manifested as menstrual thinning, hemorrhoids, hairiness, obesity and infertility. It is also characterized by abnormal levels of reproductive hormones, which can lead to anovulatory, infertile and menstrual disorders [2].

Anti-Müllerian hormone (AMH) is secreted by the granulosa cells of the pre-antral and antral follicles. AMH has drawn much attention in the field of female reproduction as a hormone that has important regulatory functions during follicular maturation. The level of AMH can reflect the number of ovarian antrum follicles, ovarian reserve, and ovarian function [3]. AMH levels in the blood are reliable biomarkers of actively growing primary to non-dominant antral follicles. In female primates, and most probably in women too, AMH does not regulate primordial follicle growth. It stimulates primary and secondary follicle growth, in contrast to its inhibitory role in female rodents. AMH, however, does inhibit FSH action on antral follicle growth, as it does in rodents [4]. Further, AMH is involved in follicular dysfunction and therefore reflects the degree of disturbance in follicular development [5–6]. Serum AMH is not affected by menstrual cycle and oral contraceptive use, so it has potential as a marker for the diagnosis of PCOS [7]. Studies have shown that the level of circulating AMH is two- to three-fold higher in women with PCOS than in healthy women of childbearing age, probably due to increased follicular mass associated with PCOS [8–9]. However, women without PCOS who have high AMH levels are commonly very fertile.

Although increase in AMH levels has been reported to be associated with PCOS, there is little consensus on the appropriate reference range. Our aim was to explore the diagnostic value of AMH and other hormones or glucose metabolism markers for detecting PCOS in Chinese women. Our hypothesis is that AMH can be used as a potential predictor of PCOS, and that it can be used in combination with other indicators to improve detection.

## 2. Materials and methods

### 2.1 Subjects

This is a retrospective study that was conducted on women who consulted our endocrinology clinic at the Obstetrics and Gynecology Hospital of Fudan University between January 2016 and October 2016. Of the 3,400 women who consulted, we selected 653 women diagnosed with PCOS from among the outpatients for our retrospective analysis. In addition, 118 other participants were selected as healthy controls after exclusion of other endocrine and gynecological diseases.

PCOS was diagnosed in the 653 participants based on the 2003 Rotterdam ESHRE/ASRM criteria: oligo and/or anovulation (OA); clinical and/or biochemical signs of hyperandrogenism (HA); and presence of polycystic ovaries (PCOs), defined as the presence of ≥12 follicles measuring 2–9 mm in diameter in each ovary and/or increased ovarian volume (>10 ml) [6]. OA was defined as a cycle length of more than 35 days or amenorrhea. Biochemical HA was defined as circulating total testosterone levels above the 95th percentile (0.51 ng/mL). Clinical HA was defined as the presence of hirsutism, acne, and/or alopecia. Hirsutism was defined as the presence of hirsutism, as evidenced by an m-FG score ≥ 8 [10].

The exclusion criteria were as follows: Cushing’s syndrome, dysfunctional uterine bleeding, primary amenorrhea, hypothalamic amenorrhea, pituitary amenorrhea, uterine amenorrhea, hyperprolactinemia, premature ovarian failure, ovarian functional tumors, theca cell proliferation, adrenal cortical hyperplasia or tumor, thyroid dysfunction, auto-immune disease, malignancy, central nervous system disease, current or previous use of oral contraceptives within 6 months of enrolment or the use of medications affecting the hypothalamic-pituitary-ovarian axis (e.g., anti-androgens, ovulation induction agents, antidiabetic medications, anti-obesity medications or glucocorticoids), age <20 or ≥40 years, and absence of complete records of hormone testing or ultrasonography. None of the participants were taking any form of steroid drugs before their samples were collected.

The present study was conducted in accordance with the tenets of the Declaration of Helsinki and received the approval of the Research Ethics Committee of the Obstetrics & Gynecology Hospital of Fudan University (26 Feb 2016, Kyy2016-22). All data were fully anonymized before they were accessed, and the ethics committee waived the requirement for informed consent.

### 2.2 Serum samples

Peripheral blood samples (5 ml) were obtained on days 2–5 of the menstrual cycle. The sample was collected in a serum separator tube and allowed to clot for 30 min before centrifugation at 1000 × *g* for 15 min. All peripheral blood samples were processed within 2 h of collection.

### 2.3 Measurement of serum hormones and metabolism indicators

Serum AMH was detected with the UNION immune analyzer’s AMH detection kit (single test strip) (YHLO, ShenZhen, China). Serum follicle-stimulating hormone (FSH), luteinizing hormone (LH), progesterone (P), prolactin (PRL), estradiol (E2), testosterone (T) and dehydroepiandrosterone sulfate (DHEA-S) were measured with Beckman Coulter DxI800. Sex hormone-binding globulin (SHBG) was measured on the Roche Cobas E601 automatic immunoassay apparatus. 17-α-OH progesterone (17α-OHP) was detected with the 17α-OH Progesterone ELISA Kit (DRG International, Germany). Fasting plasma glucose (FPG) was measured with the Hitachi 7180 automatic biochemical analyzer. Fasting insulin (INS) was measured with the Architect i2000 chemiluminescence immunoassay analyzer. Both intra-assay and inter-assay coefficients of variation were less than 10% for all the assays.

Glucose tolerance was evaluated using the criteria of the American Diabetes Association [11]. Based on fasting serum glucose and insulin, the homeostasis model assessment for insulin resistance (HOMA-IR) index was calculated with a standard formula: HOMA-IR = fasting INS (mIU/ml) × fasting glucose (mmol/l)/22.5. The free androgen index (FAI) was calculated using the following formula: total testosterone/SHBG ×100 [12].

### 2.4 Transvaginal or transrectal ultrasonography

Ultrasonography was performed for all of the participants on the same day as blood sample collection. A transvaginal or transrectal ultrasound examination was performed by senior doctors with a 4–9 MHz probe using Voluson 730 Pro (General Electric, Milwaukee, WI, USA). Measurements were obtained in real time at the early follicular phase for determination of ovarian morphology. Each ovary was scanned in both longitudinal and transverse cross-sections from the inner to the outer margins to count the total number of follicles. The volume of each ovary was determined, and all follicles with a diameter of 2–9 mm were counted in each ovary. Ovarian volume was calculated for each ovary using the formula for a prolate ellipsoid: π/6 × (D1 × D2 × D3), where D represents the maximum diameter along the transverse, anteroposterior, and longitudinal axes [13].

### 2.5 Statistical analysis

For data with normal distribution and homogeneity of variance, an independent-sample *t* test was used to compare differences between two groups, and one-way ANOVA with Tukey’s post-hoc test was performed if there were three or more means. For non-normally distributed data, differences between groups were evaluated with the non-parametric Mann-Whitney *U*-test. In order to evaluate the diagnostic effectiveness of serum AMH and metabolic parameters in PCOS patients, we used receiver operating characteristic (ROC) curves and logistic regression analysis of AMH, LH/FSH, T and INS. Two different approaches were used to determine the optimal cutoff value of AMH to classify PCOS: Youden index, which is the maximum sensitivity-(1-specificity), and the shortest distance on the ROC between the optimal sensitivity and specificity ([1 − sensitivity]^2^ + [1 − specificity]^2^) [14]. Sensitivity, specificity, positive likelihood ratio (PLR: sensitivity/[1 − specificity]), and negative likelihood ratio (NLR: [1 − sensitivity]/specificity) of the cutoffs were also calculated [15].

All statistical analyses were performed by means of GraphPad Prism version 5.0 for Windows (GraphPad Software, USA), and p < 0.05 was considered to indicate significant differences.

## 3. Results

### 3.1 PCOS phenotypes

The 653 patients were divided into four groups: OA+PCO (Type 1), OA+HA (Type 2), HA+PCO (Type 3), OA+HA+PCO (Type 4). Type 4 (characterized by all the Rotterdam criteria) is the most common type, while type 1 is the least common type. No statistical difference in age and BMI was observed between the PCOS groups and the control group (p > 0.05) (Table 1).

**Table 1:** Demographic characteristics of the PCOS patients and controls.

Phenotype	OA	HA	PCO	n	Frequency	Age	BMI (kg/m^2^)
1	+	-	+	126	19.3%	27.8±3.9	25.2±4.6
2	+	+	-	175	26.8%	27.8±4.3	24.8±6.2
3	-	+	+	163	25.0%	26.7±4.0	25.7±3.9
4	+	+	+	189	28.9%	26.9±4.2	26.2±5.2
control	-	-	-	118	/	27.3±4.1	25.5±5.6

### 3.2 Hormonal and metabolic characteristics of the PCOS patients

The hormonal and metabolic characteristics of the study patients and controls are shown in Tables 2–5. FSH was significantly lower in the type 4 patients than in the controls (p < 0.05), but no significant differences were observed between the other types and the controls. The women with PCOS (all four types) had significantly higher LH, LH/FSH, AMH, FAI%, 17α-OHP, and fasting INS than women in the control group (p < 0.05) (Tables 2–5). Polycystic ovary patients aged 20–40 years are mainly concentrated in the 20–29 years of age, accounting for 73%. The AMH level was significantly higher in PCOS patients in the age groups 20–29 and 30–39 years than in the normal control group (Table 3). Serum T, SHBG, DHEA-S and HOMA-IR levels were significantly higher in the PCOS patients than in the control group (Tables 4 and 5). No significant differences in PRL, P, E2, and fasting glucose were observed between the groups (Tables 2–5).

**Table 2:** Hormone concentrations in the PCOS patients and controls.

	PCOS n = 653	OA+PCO n = 126	OA+HA n = 175	PCO+HA n = 163	OA+PCO+HA n = 189	Normal n = 118
FSH (mIU/ml)	6.6 ± 1.4	6.8 ± 1.3	6.5 ± 1.6	6.8 ± 1.4	6.5 ± 1.3**	7.1±1.3
LH (mIU/ml)	13 ± 6.1***	10 ± 5.4***	13 ± 6.2***	13 ± 6.6***	13 ± 5.7***	7.2±3.7
PRL (ng/ml)	11 ± 4.9	11 ± 5.0	11 ± 5.3	11 ± 5.1	11 ± 4.5	12±4.6
E2 (pg/ml)	62 ± 51	50 ± 29	74 ± 56	60 ± 40	62 ± 42	65±26
P (ng/ml)	0.52 ± 0.3	0.45 ± 0.32	0.52 ± 0.29	0.54 ± 0.28	0.57 ± 0.3	0.49±0.27
AMH (ng/ml)	9.3 ± 3.1***	9.1 ± 3.0***	8.9 ± 3.2***	9.3 ± 3.5***	9.9 ± 2.9***	5.7±2.1
LH/FSH	2 ± 0.94***	1.5 ± 0.78***	2.1 ± 0.91***	2 ± 1***	2.1 ± 0.9***	1.2±0.5

** *P* < 0.01;

*** *P* < 0.001

**Table 3:** AMH levels in the PCOS patients and controls according to age.

Age group	PCOS	OA+PCO	OA+HA	PCO+HA	OA+PCO+HA	Normal
20–29 (y)						
n	477	91	117	126	143	53
Percentage	73% (477/653)	19.1%	24.5%	26.4%	30%	/
AMH (ng/ml)	9.4 ± 3.1***	9 ± 3***	9.2 ± 3.2***	9.4 ± 3.4***	9.9 ± 2.9***	6.2 ± 2.5
30–3 (y)						
n	176	35	58	37	46	73
Percentage	27% (176/653)	19.9%	33%	21%	26.1%	/
AMH (ng/ml)	8.5 ± 3.7***	8.7 ± 3.6***	7.8 ± 3.8***	8.4 ± 3.4***	9.5 ± 3.5***	5.3 ± 2.1

***P < 0.001

**Table 4:** Androgen and metabolic parameters in the PCOS patients and controls.

	PCOS n = 653	OA+PCO n = 126	OA+HA n = 175	PCO+HA n = 163	OA+PCO+HA n = 189	Normal N = 118
T (ng/ml)	0.66 ± 0.22***	0.4 ± 0.096	0.69 ± 0.14***	0.74 ± 0.23***	0.75 ± 0.2***	0.42±0.13
SHBG (nmol/ml)	60 ± 53*	63 ± 56	70 ± 58	44 ± 35***	60 ± 54*	66±39
FAI (%)	7.5 ± 6.3***	4.6 ± 4.2*	6.3 ± 5.6***	10 ± 7.1***	8 ± 6.3***	2.6±1.6
DHEA-S (μg/dl)	285 ± 103***	207 ± 75	302 ± 99***	315 ± 105***	295 ± 97***	209±63
17α-OHP (ng/ml)	0.54 ± 0.44***	0.4 ± 0.38***	0.54 ± 0.49***	0.54 ± 0.36***	0.61 ± 0.44***	0.17±0.13

*P < 0.05;

***P < 0.001

**Table 5:** Glucose metabolism parameters in PCOS patients and controls.

	PCOS n = 653	OA+PCO n = 126	OA+HA n = 175	PCO+HA n = 163	OA+PCO+HA n = 189	Normal N = 118
INS (μU/ml)	13 ± 8.2***	12 ± 6.9***	11 ± 6.2***	13 ± 8.7**	14 ± 9.3***	6.8±2.5
FPG (mmol/L)	4.9 ± 0.73	5.1 ± 1.2	4.9 ± 0.53	4.9 ± 0.59	4.9 ± 0.61	4.8±0.33
HOMA-IR	3 ± 2.4**	3 ± 2.6*	2.3 ± 1.4	3.7 ± 3.3*	3.3 ± 2.6***	1.8±1.5

*P < 0.05;

** *P* < 0.01;

***P < 0.001

### 3.3 AMH and hormone and metabolic indicators of PCOS

For the ROC curves for AMH as a predictor of PCOS, we grouped the subjects into two age groups: 20–29 years and 30–39 years. The area under the curve (AUC) was 0.846 (95% confidence interval: 0.815–0.864) for AMH as a predictor of PCOS in the 20–29 years age group. The cut-off level of AMH was 8.16 ng/ml, which had a sensitivity of 78.4% and specificity of 80.9%. In the age group 30–39 years, the AUC was 0.865 (95% confidence interval: 0.791–0.887), and the cut-off level value of AMH was 5.89 ng/ml (sensitivity, 82.6%; specificity, 79.8%).

For the ROC curves of the other metabolic parameters, the AUC, 95% confidence intervals, cut-off values, sensitivity, specificity, PLR, and NLR are summarized in Table 6.

**Table 6:** ROC curve values for all the variables individually and in combination.

	AUC	95% CI	Cut-off value	Sensitivity	Specificity	PLR	NLR
AMH (20–40 y) AMH (20–29 y) AMH (30–39 y)	0.854 0.846 0.865	0.826–0.872 0.815–0.864 0.791–0.887	>7.69 >8.16 >5.89	74.3 78.4 82.6	81.6 80.9 79.8	3.69 3.75 3.54	0.35 0.33 0.25
LH	0.784	0.754–0.806	>9.83	65.1	85.2	4.34	0.43
LH/FSH	0.795	0.762–0.826	>1.3	69.8	82.7	3.65	0.35
T	0.813	0.785–0.838	>0.5	81.2	66.8	2.58	0.28
FAI%	0.805	0.761–0.847	>3.7	67.9	86.9	5.94	0.36
INS	0.75	0.718–0.802	>9.2	58.2	86.5	4.36	0.47
HOMA-IR	0.722	0.63–0.759	>1.5	70.1	65.8	2.45	0.42
AMH+L/F+T+I	0.951	0.922–0.954	/	/	/	/	/

With AMH, LH/FSH, T and INS as independent variables, a logistic regression model was established, in which the ROC curve for all the variables together was fitted with the probability value of the model. The area under the ROC curve was 0.951 (95% confidence interval: 0.922–0.954). The area under ROC curve for all the variables combined was significantly greater than that for each of the variables separately.

### 3.4 Correlation between AMH and other biochemical variables

The concentration of AMH was found to be significantly associated with FSH, LH, LH/FSH, T, and ovarian volume changes in PCOS patients. However, the AMH concentration was not significantly correlated with FAI%, INS or HOMA-IR (Table 7).

**Table 7:** Correlation of AMH concentration with biochemical variables.

	r	95% CL	*P* value
FSH	-0.19	-0.26 to -0.11	<0.0001
LH	0.28	0.21–0.34	<0.0001
LH/FSH	0.32	0.26–0.39	<0.0001
T	0.33	0.26–0.4	<0.0001
FAI%	0.05	/	0.43
INS	0.11	/	0.1
HOMA-IR	0.1	/	0.15
LOV	0.3	0.22–0.37	<0.0001
ROV	0.29	0.22–0.36	<0.0001

## Discussion

This study reports the reference range of AMH for the diagnosis of PCOS in the Chinese population. It also confirms the diagnostic value of other known metabolic and hormonal markers of PCOS in this population.

Wiweko et al. reported that AMH levels are higher in PCOS when hyperandrogenism is present [16], which is different from our findings. Our finding showed that compared to healthy controls, all the women with PCOS, even those who did not have PCOM or HA, had significantly higher AMH levels. This is probably because the AMH level in patients with PCOS is not only related to increase in the follicle pool but also increase in the production per follicle [9,17]. Another explanation might be that the ultrasound findings were false negatives. In fact, Dewailly and colleagues have shown that excessive serum AMH levels are found in most cases with false negative ultrasound findings. Accordingly, when both markers are applied, phenotype 2 virtually disappears. Phenotype 2 may be therefore be an artifact that leads to poor detection of PCOS [18].

Because AMH can reflect the number of antral follicles, it might be easier and less expensive to use a blood test than ultrasonography in cases where it is not convenient to conduct an ultrasound examination. Using both the AMH and ultrasound findings would probably increase the diagnostic sensitivity for PCOS.

Consistent with previous studies, our findings showed significantly higher AMH levels in the PCOS patients than in the controls. In addition, in the PCOS patients in the current study, the FSH level was slightly decreased and the LH level was significantly increased. It is possible that AMH affects follicular growth by suppressing the expression of the aromatase-dependent LH receptor [9]. High levels of AMH lessen the sensitivity of follicles to FSH, which results in defective selection of the lead follicle and culminates in anovulation and relative stockpiling of growing follicles [19–20]. LH strengthens this effect, because when granulosa cells are cultured with LH, the expression of AMH is up-regulated in anovulatory polycystic ovaries, which is not observed in normal ovaries [21]. This is supported by the significant correlation observed between AMH and FSH, LH, and LH/FSH, which is in agreement with previous reports [9,17, 22,23].

The intra-ovarian environment, especially the androgen concentrations, is believed to be involved in the pathophysiology of PCOS. Testosterone assays, however, are notoriously inaccurate for women. Therefore, the testosterone assay has limited accuracy for PCOS diagnosis. In addition, a study by Dewailly et al. indicated that AMH may be an alternative indicator of classical hyperandrogenism [24]. Our study found a significant correlation between AMH and T, which is in agreement with previous reports [22, 25]. With regard to the underlying pathway, Jonard et al. have reported that intra-ovarian hyperandrogenism may damage the ovulation cycle by preventing the cyclical increase of FSH via AMH production by granulosa cells. Another consequence of hyperandrogenism would be hyperinsulinism or insulin resistance, which would in turn amplify the intra-ovarian hyperandrogenism [23].

Women with PCOS are commonly insulin resistant (up to 75% of lean and 95% of overweight women with PCOS) and are two to four times more likely to be obese and develop type 2 diabetes [26–27]. Our findings showed that the fasting insulin and HOMA-IR levels were significantly higher in patients with PCOS (except for those with Type 2 PCOS) than in controls. The optimal sensitivity and specificity of 71.1% and 69.7% respectively were found at a HOMA-IR cut-off level of 1.6.

Although increased AMH in PCOS has been reported, there is little consensus on an appropriate reference range. In addition, a significant mean difference was observed between the reference range determined with Access Dxi or Cobas and EIA AMH/MIS ELISA; this contradicts a previously published study that compared AMH assays. It reported with the help of ROC analyses in various literature reports that each assay displayed similar efficiency for PCOS diagnosis, with the reported sensitivities varying from 49% to 74% when the specificity was set at 92% [28].

We found that AMH levels fall with increasing adult age in women with PCOS, as well as in women without PCOS. In both younger and older women, nevertheless, AMH levels in women with PCOS usually exceed those in women of comparable BMI who do not have PCOS. Therefore, it is necessary to set up different threshold values for PCOS diagnosis using different AMH assays and for different age groups. Another new aspect of our findings is that AMH levels were significantly higher than the controls even in type 1 and 2 of PCOS. Finally, given that ultrasound examination may yield false negative results, AMH examination could be used to ensure that PCOS is not missed in such cases.

## Conclusion

Our results show that AMH may have potential as a marker of PCOS. The cut-off level of AMH for diagnosing PCOS was 8.16 ng/ml in the 20–29 years age group and 5.89 ng/ml in the 30–39 years group in the current study population from China. The results also indicate that serum AMH, LH/FSH, T and INS can be used for the diagnosis of PCOS; in particular, combined detection with these markers could improve the diagnostic specificity and sensitivity of PCOS.

## Supporting information

S1 Data Data. (XLSX)

S2 Data Data in English. (XLSX)
